# Human Serum Eye Drops in Eye Alterations: An Insight and a Critical Analysis

**DOI:** 10.1155/2015/396410

**Published:** 2015-10-04

**Authors:** Maria Rosaria De Pascale, Michele Lanza, Linda Sommese, Claudio Napoli

**Affiliations:** ^1^U.O.C. Immunohematology, Transfusion Medicine and Transplant Immunology, Regional Reference Laboratory of Transplant Immunology, Azienda Ospedaliera Universitaria (AOU), Second University of Naples, 80100 Naples, Italy; ^2^Multidisciplinary Department of Medical, Surgical and Dental Sciences, Second University of Naples, 80100 Naples, Italy

## Abstract

Human serum contains a physiological plethora of bioactive elements naturally released by activated platelets which might have a significant effect on the regeneration of corneal layers by stimulating the cell growth. This mechanism supported the use of human serum eye drops in some ocular diseases associated with dystrophic changes and alterations of the tear film, such as persistent corneal epithelial defects and dry eye syndrome. We focused our effort on potential benefits and limitations of the use of human serum eye drops when conventional therapies failed. We reviewed the recent literature by reporting published studies from 2010 to 2014. Despite the limited evaluated study populations, most of the clinical studies have confirmed that serum eye drop therapy is effective in corneal healing by reducing ocular symptom, particularly during the short-term follow-up. In addition, three recent published studies have shown the efficacy of the serum eye drop therapy in comparison to traditional ones in intractable patients. Besides, reported ongoing clinical studies confirmed the open debate regarding the use of biologic tools for cornea regeneration. Results from these studies might open novel challenges and perspectives in the therapy of such refractory patients.

## 1. Introduction

**Figure 1:**
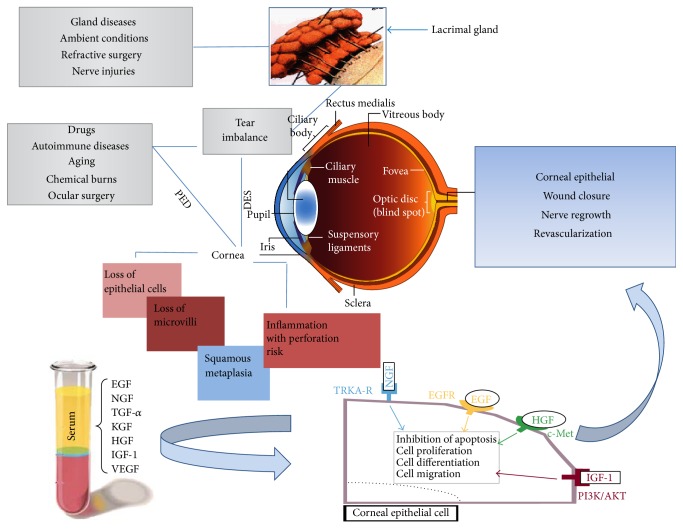
Mechanisms involved in damaged cornea and serum growth factor regeneration pathways. Several corneal injures can promote pathological conditions such as PED or DES. Action of drugs, autoimmune diseases, aging, chemical insults, and postsurgical lesions can wound the cornea directly or indirectly through the imbalance of the lachrymal gland. Microscopically, these pathogenic conditions result in a loss of epithelial cells and microvilli, epithelial squamous metaplasia, and inflammation of the corneal surface. The corneal healing process can benefit from the promoting action of serum GFs. Once they are released, through tyrosine kinase receptors, they propagate their signal from the plasma membrane to the nucleus. Through explicit pathways and signaling cascades, GFs activate the expression of target genes involved in apoptosis, proliferation, cell differentiation, and migration. This synergistic action establishes regenerative effects with closure of epithelial lesions, revascularization, and neurorepair.

Cornea is mostly composed of collagen and water and is enveloped by epithelium and endothelium [1]. These layers cooperate to ensure tissue homeostasis by providing adequate corneal transparency and reliability [1]. After injury, corneal epithelial cells regenerate and restore the physiologic tissue architecture. In addition, a concomitant nerve regrowth and a controlled neovascularization of the damaged surface may occur [2, 3]. Cellular loss needs replacement by cell growth and migration [3]. The mechanism driving the epithelialization involves a multiplicity of cells stimulated by serum growth factors (GFs) (Table 1) [4–6], mostly contained in platelet-*α* granules and issued by the same GFs into the blood during stress and tissue repair [4–10]. The great quantity and accessibility of GFs and other signaling proteins in platelets with a consequent inhibition of cell apoptosis and improvement of cell proliferation, differentiation, and migration suggested the extensive use of platelet derivatives for clinical and surgical aims in regenerative medicine (Table 1 and Figure 1) [7, 11]. Indeed, GFs, binding to tyrosine kinase or G protein-coupled receptor families, drive both the inflammatory process and the stroma remodeling through autocrine, juxtacrine, or, most commonly, paracrine means. Thus, the transcription of critical proteins for cell cycle returning to prewounding levels after the tissue healing occurs (Table 1) [12, 13].

Particularly, serum GFs such as epidermal growth factor (EGF), hepatocyte growth factor (HGF), and keratinocyte growth factor (KGF) stimulate corneal wound closure accelerating the healing time. Moreover, transforming growth factor-*β*1/*β*2 (TGF *β*1/*β*2) induces myofibroblast from fibroblast differentiation coupled to corneal opacification (corneal haze) (Table 1) [14–16]. Moreover, cytokines derived from the trigeminal nerve like substance P, neuropeptide y, catecholamines, and acetylcholine are positively involved in corneal healing [17]. Besides, an aged decreased response to mitogens mediated by alterations in the expression and activity of cyclin-dependent kinase inhibitors (p27KIP1, p16INK4A, and p21CIP1) appears to be involved in a lacking or a damaging of cellular repair processes [3].

Toward this context, the lachrymal film plays a critical role such as resource of GFs [18–24] since the lack of tear epitheliotropic support promotes corneal opacity onset with consequent visual impairment [25]. On the other hand, tear upregulation drives corneal epithelial hyperplasia, excessive deposition of extracellular matrix, and hypervascularization with cornea conjunctivalization [8]. Here, we report the different concentrations of each GF in the human serum with respect to tears. The levels of transforming growth factor *α* (TGF-*α*), hepatocyte growth factor (HGF), TGF*β*1/2, and nerve growth factor (NGF) resulted to be even more elevated in serum than in tears.

Failure of the corneal repair mechanisms leads to a chronic pathologic condition as persistent epithelial defects (PED) or dry eye syndrome (DES) [15]. PED result from several factors such as aging, chemical burns, systemic disorders, and drugs [26] (Figure 1). Nevertheless, DES, associated to tear deficit or tear inefficiency, is able to promote the corneal epithelial instability and inflammation [27, 28] supporting PED syndrome. DES is caused by lacrimal gland imbalance often connected to systemic inflammatory diseases [29–32], such as Sjogren's syndrome [33], rheumatoid arthritis [31], diabetes [34], systemic lupus erythematosus, acne rosacea, and Graves' disease [35]. In addition, hormonal modifications, drugs (e.g., systemic antihistamines, diuretics, and topical beta blockers for glaucoma therapies), and surgeries (e.g., photorefractive keratectomy and laser* in situ* keratomileusis) [36] as well as the repeated use of contact lenses could be involved in DES development [27]. On the basis of mechanistic criteria, International Dry Eye WorkShop has characterized two main subtypes of the disorder (aqueous deficiency and evaporative dry eye) both interested by tear film instability and symptoms of discomfort [28, 37]. Ocular dryness or irritation might increase light sensitivity, foreign body sensation, red eyes, poor vision, and daily life limitations which are the most referred symptoms which have great impact on patient quality of life [37, 38]. The best clinical marker for DES diagnosis and for the severity assessment is represented by the improved tear osmolarity [39]. In addition, tear production is currently evaluated by Schirmer's testing, fluorescein clearance, and fluorescein tear break-up time (TBUT). The ocular surface damage is estimated through dye staining (fluorescein and lissamine green) while the severity of subjective symptoms is assessed by subjective scored questionnaires (like OXFORD score and Ocular Surface Disease Index) [37, 38].

Up to now, there is no gold standard therapy for DES or PED [40–42]. Current therapeutic strategies require the accurate identification of etiologic mechanisms that cause the corneal injury by providing epitheliotropic factors and enhancing tear replacement [26, 43, 44]. When standard therapeutic options fail, the main treatment purpose is the increased patient comfort and corneal moisture through the instillation of artificial tears, corticosteroids, antibiotics, and use of bandage contact lenses [30, 45–47]. However, natural tears have a particular composition of water, salts, hydrocarbons, proteins, and lipids that cannot be restored by pharmacological alternatives [25]. Furthermore, artificial tear substitutes contain chemical preservatives associated with toxic and allergic reactions, especially for those patients with sensitive eyes [6]. Moreover, the repeated instillation of topical corticosteroids could be associated with long-term side effects including cataracts and increased intraocular pressure [48]. For these motivations, alternative therapies like silicone punctal plug insertion, botulinum neurotoxin type A, nutritional supplements (essential fatty acids, including omega-3, linoleic acid, and gamma-linoleic acid), and topical 0.05% solution of cyclosporine A have been proposed [49–55]. However, changes in life style as an increased water intake and reduction of alcohol consumption, indoor humidifiers, and air filters or cleaners have been recommended [56].

A debated aspect of the treatment of corneal diseases is focused on the use of novel regenerative instruments for corneal regeneration [57–60]. The evidence regarding the key role of several GFs for the integrity of the ocular surface (Table 1) fits the use of single recombinant GFs in several human corneal degenerative disorders [57–61]. Nerve growth factor (NGF) alteration in corneal diseases has been largely evaluated; NGF pathway alteration has been tested in an animal model by demonstrating NGF to be involved in corneal healing and in sensory denervation [62]. Moreover, in studies evaluating human being, low tears level of NGF has been proved to be reduced in eyes affected by dry eye [63] and has been proved to be effective in several corneal diseases such as neurotrophic keratitis, immune corneal ulcer, and HSV keratitis and after cataract surgery [64]. Clinical trials are ongoing to evaluate therapy with NGF eye drops in corneal diseases and first results seem to be very promising [65].

In addition, a conditioned medium derived from human uterine cervical stem cells has been tested for corneal epithelial healing [66], and a therapeutically ocular surface medium, routinely used to culture epithelial cells, was suggested as novel eye drops for DES and PED [67].

Among these emerging therapies, the use of biologic eye drops derived from both human peripheral [44, 50, 68–75] and umbilical cord blood serum [76–78] plays a crucial role in several corneal diseases. Previous* in vitro* experiments showed that corneal epithelial cell morphology and cell functions are better maintained by human serum eye drops (SE) than pharmaceutical tear substitutes [79].

The first applications of human SE to support corneal regeneration were performed in 1975 in corneal alkali injury cases [80]; later, in 1984, Fox et al. [81] reported the use of SE in a DES. Later, SE have gained a therapeutic dignity in ophthalmology as a new concept to manage wounded cornea [82, 83]. To date, despite the fact that SE therapy could avoid drug side effects, its use is restricted and is not universally recognized as therapeutic option although several aspects of the whole regenerative medicine are still debated [11].

Here, we critically analyzed the current applications of SE in corneal diseases like DES and PED by focusing on crucial topics for its production and the current legislative restrictions in support of its use. To analyze the SE therapeutic achievement, we reported the most recent published randomized clinical trials (RTCs) and ongoing studies where this kind of treatment has been applied and compared to standard and other emergent treatments in severe ocular conditions.

## 2. Legislative, Ethical, and Technical Implications on the Use of Serum Eye Drops

The use of SE obtained from patient peripheral whole blood (autologous) or from healthy donor (nonautologous or allogenic) represents a biological therapeutic strategy influencing and promoting the corneal restitution [11, 68, 83]. The rationale for its use arises from its strong similarity to tears, in regard to pH (7.4) and osmolarity (298 mml for tears and 296 mml for serum) [84] as well as its biochemical constitution. In addition, SE offer the same platelet derived antibacterial and anti-inflammatory effect* in vivo* [85].

Despite the fact that multiple studies supported the safety and efficacy of SE over standard treatments, SE have not yet been considered for approval by Food and Drug Administration, in the United States [8].

For this reason, SE are not a recognized treatment and are not covered by most of medical insurances [82]. In addition, despite the improved reliability of current serologic tests for HBs-Ag, anti-HCV, anti-HIV-1/2, and syphilis detection, the use of allogenic SE is associated with the immunologic and infectious implications of donor exposure [86]. However, even though autologous SE should be considered the best choice, patients with absolute contraindications to provide blood as a result of specific diseases or conditions (e.g., bacteremia) or who are unable to tolerate frequent venipunctures are eligible for the treatment with healthy donor SE. The allogenic SE, subjected to severe laboratory checks, can be used as an effective alternative treatment showing comparable clinical results to the autologous one [73, 86].

The established criteria for donor enrollment and for blood collection include hemoglobin level higher than 11 g/dL (hematocrit > 33%) and exclude subjects presenting risk of bacteremia and cardiovascular diseases deferring pregnant women and children. Despite the absence of absolute prohibitions, the use of SE in children is restricted to avoid repeated required venipunctures and limiting instillation of potentially infectious donor sera in such patients [86, 87]. In these cases, if traditional therapy with artificial tears, antibiotics, or steroids is not effective, the topical cyclosporine or the conjunctival flap can be applied to improve the corneal conditions [87].

According to the current protocols, blood samples should be collected with previous informant consent and transferred into a sterile kit or blood bag without anticoagulant and treated under sterile conditions (laminar flow hood) (Table 2). A sufficient time has to be dedicated to the patient to illustrate properly the treatment and the need for repeated blood sampling. As shown in Table 2, the routine production of SE is frequently affected by the lack of recognized procedures [11, 85]. However, the proper management, handling, and storage of final product are essential for the successful treatment avoiding side effects.

To date, many laboratory protocols have been published for SE production with variable dilutions of serum (from 20% to 100%) and with differences in clotting phase, centrifugation time, and speed [88, 89] (Table 2). According to previous studies, clotting time is critical to obtain ideal concentrations of EGF, TGF-*β*-1, and fibronectin in the collected serum [85]. In addition, a proper serum dilution is recommended to reduce the antiproliferative effect of TGF-*β* which results five times higher in human serum than in tears [85]. Usually, 0.9% sodium saline or balanced salt solutions are utilized as diluents and preservatives solutions are not usually added to decrease the risk of induced toxicity. For the lack of preservatives, microbial cultures as well as the antibiotic therapy are recommended [85]. Finally, to preserve the biologic activity, the SE should be frozen in blood banks at −70°C until its use or at −20°C for a month and protected from light to prevent the degradation of vitamin A (Table 2).

Depending on clinical conditions, the frequency of instillations of SE may go from every 15 minutes to two times per day; also the duration of the reported treatment widely ranged from 3 days to a maximum of 36 months. In most cases, the improvement appears after a brief period of therapy (from 1 to 4 weeks) [90]. Studies reported one case of corneal immunoglobulin deposition, scleral vasculitis, epithelial erosion, conjunctivitis, decreased corneal sensitivity, inflammatory response, infections, and an isolated case of mycosis [90]. Recently, to avoid immunity complications, Anitua et al. have characterized a plasma rich in GF without IgE and complement suggesting its use in autoimmune diseases [82]. Until now, the few published data on clinical complications associated with SE have demonstrated the safety of this therapy. However, limitations in published studies as discussed below are observed.

## 3. Clinical Applications and Study Results

As shown in Table 2, several studies investigated the role of SE treatment mostly in corneas affected by DES and PED.

Despite the fact that most of the authors found objective and subjective wellbeing after the treatment with SE, the comparison of clinical results is complex because data have been obtained from nonhomogeneous populations affected by several unrelated corneal diseases. In addition, the technical preparation of SE shows different dilutions obtained with different solutions, clotting phases, centrifugation forces, and time intervals, as well as different storage temperatures and times that can modify the final clinical outcomes and healing times as shown in Table 2.

Recent results have confirmed the efficacy of SE with respect to conventional therapy [91] in patients with severe DES or PED [79, 92] both by improving tear film stability and providing subjective comfort. Moreover, two recent prospective interventional studies [93, 94], on large cohorts of patients with PED treated with SE after ocular surgery, showed a significant or moderate improvement of delayed heal. In particular, Chen et al. [93] showed that 165 patients treated with 20% SE after penetrating keratoplasty drastically reduced postoperative PED when compared to patients that received artificial tears. In addition, Lekhanont et al. [94] evaluated SE for PED in 181 patients showing a high proportion (93.92%) of complete corneal epithelialization in only 4 days with low rate of adverse reactions.

Case reports have been described about the use of SE in other corneal diseases like ocular graft versus host disease [95, 96], bullous keratopathy [97], fulminant bilateral* Haemophilus influenzae* keratitis [98], neurotrophic corneal ulcer [99], anterior tissue necrosis after porous orbital implant [41], and Mooren's ulcer [100]. In all these cases, SE allowed a complete corneal healing with an effective improvement of the clinical conditions.

Despite many promising results, some recent studies have questioned the validity of this treatment. A prospective cross-sectional study on 34 patients did not find that SE could be effective in secondary Sjogren's syndrome due to elevated serum proinflammatory cytokine levels [101]. Moreover, a single prospective study on 17 patients with DES demonstrated the short-term benefit of SE, which persisted up only to three months after the end of therapy [102]. In 2013, Pan et al. [36] performed a meta-analysis identifying four randomized clinical trials, which compared SE with artificial tear treatment or saline solutions in patients with Sjogren's syndrome-related DES, non-Sjogren's syndrome DES, and postoperative DES. In conclusion, they advocated the need of recognized measures to define subjective symptoms and to assess the real effect of SE therapy for DES [36]. The use of SE was compared in randomized trials to unconventional biologic therapies, which have gained a growing interest, such as umbilical cord blood serum (CBS) [103, 104] and amniotic membrane transplant [105]. CBS is collected from umbilical vein after fetal delivery, manipulated, and collected similarly to peripheral serum [104]. CBS is considered a reliable source of undifferentiated mesenchymal stem cells [106, 107], which are self-renewal elements that are able to replace directly corneal keratocytes [104] and conjunctival, limbal [76], and retinal nerve cells [104]. In addition, CBS contains consistent levels of cytokines, GFs, fibronectin, prealbumin, and fatty oils that provide useful instruments for corneal differentiation [74, 108]. Moreover, CBS includes antibacterial agents as IgG, lysozyme, and complement but lower levels of vitamin A compared to peripheral serum [109]. Despite the reduced immunogenicity of CBS with respect to peripheral serum due to the lower levels of IgM anti-A, anti-B, and IgG2 [78, 107], the use of the first is naturally associated to donor exposure and increased infectious risk and subjected to obstetric factors which could modify GF levels [110].

Some authors tested CBS for PED [77, 103], DES [111], corneal diseases due to chemical burns [112], and surgeries associated [113, 114]. Due to prominent GFs, anti-inflammatory cytokines, and mesenchymal stem cell levels in CBS, some authors demonstrated the main effectiveness of CBS with respect to both conventional treatment and traditional SE in severe corneal diseases [77, 104, 109, 112]. For this reason, clinical ongoing trials are mostly focused on the comparative evaluation of the use of SE and CBS, especially in DES, PED, and ocular GVHD as summarized in Table 3. In six ongoing studies reported on CBS eye drops, four are comparing CBS* versus* traditional SE and only one study analyzes amniotic membrane transplant* versus *SE in 180 patients with PED. Probably, promising results will arise from a large cohort randomized intervention single blind study which has enrolled 165 patients with diabetic retinopathy and penetrating keratoplasty comparing SE with standard treatments like corticosteroids and antibiotics. To date, there are no follow-up studies on SE or CBS eye drops treatment.

## 4. Future Directions

Several fields of medicine are focusing on a regenerative approach to treat pathologic conditions affected by insensitivity and toxic reactions to standard therapies. In this context, tissue engineering and regenerative medicine are the present and the future aim of clinical therapy, especially where traditional treatments fail or promote severe adverse events.

A number of corneal conditions are often not fully managed by standard treatments and are characterized by intolerances and systemic effects. New treatments have to be considered. Subjective and objective results suggest that biologic therapies for corneal surface alterations like SE treatment could be an effective option. Indeed, the use of biologic eye drops provides the beneficial effects of vitamins, GFs, and cytokines by correcting delayed corneal healing pathways and by restoring balanced mechanisms [76, 78, 79].

However, the technical preparation of human serum for ocular instillation should require a well-equipped laboratory with specialized trained personnel as well as the respect of aseptic and quality procedures. In addition, methods for SE production (clotting time, centrifugation, and concentration) including the proper additive and GF doses should be optimized according to well-established guidelines and standardized quality controlled protocols [9, 36, 111]. Specifically, a proper serum dilution should be performed to reduce the TGF *β*1/*β*2 levels (present in more than 5 times in serum compared to tears), which would promote corneal scar formations and a delayed reepithelialization (Table 1) [113]. Additionally, informed consent should be obtained from each patient in case of allogenic somministration to avoid ethical and juridical implications owing to blood transfusion practices and legislative restrictions should be carefully respected to minimize the immunological and infectious risks [11, 84].

## 5. Conclusions

To date, clinical benefits of SE therapy have been demonstrated by some published studies. Most of the recent analyzed trials have tested the clinical results coming from SE treatment through the comparison with traditional therapeutic approaches such as artificial tears, antibiotics, or corticosteroids [79, 91, 92]. Several randomized studies suggest that SE treatment leads to an improved tear film stability and subjective comfort [29, 69, 83, 88, 91, 93, 94] by determining a faster epithelial healing time and a better corneal transparency without increase of vascularization or fibrosis. Moreover, several data have confirmed the safety and the almost absolute absence of toxic and side effects, especially in severe case of DES or in PED (except those related to an improper handling). A critical point of these published studies concerns the number of patients enrolled which are recurrently less than 100 [4, 13, 16, 35, 38, 39, 43, 84, 85, 95, 96] and the almost absence of long follow-up studies [69]. On the other hand, the evaluation of the ongoing studies on this therapy showed that the newer fields of clinical research are focusing on alternatives to SE like CBS. In this regard, many studies are testing CBS and its therapeutic properties and safety. However, further studies with large populations comparing biological therapies with the traditional ones in corneal diseases are needed to provide the best treatment tailored to the singular patient.

Forthcoming conclusions will guide future efforts useful to clinical advances. They will clarify the therapeutic limits and resources of these emergent biologic therapies for corneal surface alterations, especially for refractory patients.

## 6. Method of Literature Search

We performed a computerized literature search on studies and trials by using the following search terms in various combinations: serum eye drops, cord blood serum, dry eye, and persistent epithelial defects. This search was achieved without any time and language restrictions in the following databases: PubMed, http://www.controlled-trials.com/, https://www.clinicaltrialsregister.eu/, https://eudract.ema.europa.eu/, and https://www.clinicaltrials.gov/. The complete reference list of the most relevant studies was compared for the methodology of serum eye drops collection, preparation, and storage. Clinical trials reported in Table 2 were selected from studies published in the last 5 years (2010–2014).

## Figures and Tables

**Table 1:** Main growth factors involved in corneal epithelial healing.

Growth factors	Utilized pathway	Cellular target	Cellular effects	Corneal effects	Tear level	Serum level	References
EGF	EGFR/erbB1/HER1, erbB2/HER2/neu, erbB3/HER3, erbB4/HER4	Corneal epithelial cells (limbal region)	It increases epithelial migration and proliferation, inhibits corneal epithelial terminal differentiation, and upregulates activations of B4 integrins.	It stimulates epithelial wound closure accelerating the healing time.	2053 ± 312.4 pg/mL	199.74/±64.74 pg/mL	[16]

TGF-*α*	EGFR/erbB1/HER1, erbB2/HER2/neu, erbB3/HER3, erbB4/HER4	Corneal epithelial cells	It increases epithelial migration and proliferation and inhibits the expression of keratin K3.	It leads edge extension in epithelial sheet migration during eyelid closure.	84 ± 19 pg/mL	120 to 207 pg/mL	[16, 20, 21]

KGF	Ras-MAPK, PI3K/p70S6	Corneal epithelial cells	It increases epithelial cell proliferation protecting them from hypoxia.	It stimulates epithelial wound closure accelerating the healing time in the limbus zone.	Not provided	10.63 ± 4.98 pg/mL	[56]

HGF	c-Met, Ras-MAPK, PI3K/AKT, p70S6K, EGFR	Corneal epithelial cells, fibroblasts	It increases epithelial cell migration and motility and proliferation and inhibits apoptosis.	It stimulates epithelial wound closure.	200 pgmL^−1^	573.9 ± 142.8 pgmL^−1^	[18, 57]

IGF-1	PI3K/AKT	Corneal epithelial cells, fibroblasts	It increases epithelial cell proliferation, inhibits apoptosis, increases chemotaxis, and increases the expression of connexin 43 in corneal fibroblasts.	It stimulates epithelial wound closure improving gap-junctions.	Not provided	173.5 ng/mL	[6]

TGF *β*1/*β*2	TGF *β*-RI TGF *β*-RII	Corneal epithelial cells, fibroblasts (limbal and central region)	It inhibits epithelial cells proliferation, increases keratocyte proliferation, and promotes myofibroblast differentiation.	It promotes scar formations, delays reepithelialization, and inhibits angiogenesis.	10 ng/mL	50 ng/mL	[24]

PDGF	PDGF-R	Endothelial cell, fibroblasts, epithelial cells	It increases endothelial cell proliferation, enhances fibroblast migration, and increases chemotaxis of epithelial cells.	It stimulates epithelial wound closure.	95–1330 ng/L	1.70 ng/mL	[22]

FGF-2	FGF-R, heparan sulfate proteoglycans, RTKs	Corneal epithelial cells, fibroblasts (Bowman's and Descemet's membrane)	It increases epithelial, endothelial, and stromal cell proliferation.	It stimulates epithelial wound closure and improves gap-junctions.	Not provided	8.3 ± 1.75 pg/mL	[13]

NGF	TRKA-R	Corneal epithelial cells, endothelial cells, fibroblasts	It increases epithelial cell proliferation and differentiation.	It stimulates epithelial wound closure, improves nerve regrowth, and induces inflammation and vascularization.	8.3 +/− 4.7 ng/ml	18.5 +/− 6.1 ng/mL	[19, 62, 63]

EGF: epidermal growth factor; TGF-*α*: transforming growth factor *α*; KGF: keratinocyte growth factor; HGF: hepatocyte growth factor; IGF-1: insulin growth factor 1; TGF*β*1/*β*2: transforming growth factor *β*1/*β*2; PDGF: platelet derived growth factor; FGF-2: fibroblastic growth factor 2; NGF: nerve growth factor.

**Table 2:** Published studies on serum eye drops from 2010 to 2014.

Type of component	Study design	Year	Patient number	Corneal disease	Clotting phase	Centrifugation/time	Dilution	Storage	Results	References
100% AS	Single-center prospective interventional study	2013	181	Corneal epithelial defects after ocular surgery	2 h at RT	3000 g/15 min	None	−20°C three months	It improves corneal healing.	[94]

50% AS	Single-center prospective study	2014	28	Acute and chronic eye pathologies	24–48 h at 4°C	4000 g/10 min	Sterile saline solution	−30°C six months −20°C three months	It stabilizes and improves signs and symptoms in eyes previously treated with conventional therapy.	[91]

20% AS, CBS	Double-blind prospective randomized controlled clinical study	2011	33	Ocular chemical burns of grades III, IV, and V	Not provided	1800 g/10 min	Sterile balanced salt solution	−20.0°C until use	Umbilical cord serum therapy is more effective than AS eye drops or artificial tears in ocular surface restoration after acute chemical injuries.	[112]

20% AS versus AMT	Retrospective study	2014	42	Neurotrophic keratitis Corneal ulcers	2 h	3000 g/15 min	Sterile saline solution	Not provided	Amniotic membrane transplantation is more effective than AS in deep corneal ulcers with postherpes neurotrophic keratitis.	[105]

100% AS	Not provided	2013	10	PED	2 h at RT	3000 g/15 min	None	4°C	Improvement in corneal healing.	[115]

100% AS	Descriptive prospective observational study	2011	15	Various ocular surface disorders	None	10000 rpm/10 min	None	−20°C three months	Redness, burning, sharp pain, and tired eyes improved in 100% of the patients, whereas dryness and sandy/gritty sensation improved in 92% of the patients.	[69]

20% AS, 20% no AS	Prospective interventional study	2010	165	PED after PK	30 min	1500 rpm/5 min	Sterile saline solution	−4°C	AS improves healing in patients with potentially delayed epithelial heal.	[93]

20% AS	Prospective, double-blind randomized crossover study	2014	40	Severe DES	2 h at RT	2600 g/10 min	Sterile saline solution	−20°C	AS eye drops are more effective than conventional eye drops for improving tear film stability and subjective comfort in patients with severe DES.	[92]

100% AS versus normal saline/ artificial tears/ antibiotics AS	Randomized study	2013	85	DES/SS, DES/no SS, PED	5 min at RT	3000 g/5 min	None versus 50% saline or artificial tears or antibiotic	Not provided	AS was the most effective in decreasing symptoms, corneal epitheliopathy, and promoting fast closure of wound.	[88]

50% AS	Prospective cross-sectional study	2014	34	SS	2 h at RT	3000 g/15 min	Sodium hyaluronate	−70°C until use	AS might not be effective for the treatment of secondary SS because of elevated serum proinflammatory cytokine levels.	[101]

20% AS	Three-month prospective study	2014	17	Severe DES	Not provided	3000 g/15 min	Isotonic buffered saline solution	−20°C four months 4–8°C one week	The positive effect of AS decreased with time but still persisted up to three months after the end of therapy.	[102]

20% AS	Double-blind prospective randomized controlled clinical study	2011	32	Acute chemical burns	Not provided	180 g/10 min	Sterile balanced solution	−20°C until use	Umbilical cord serum was more effective than AS and artificial tears in ocular surface restitution.	[112]

20% AS versus PRP	Retrospective review	2012	28	DES	Not provided	3000 g/15 min 200 g/11 min	Sterile saline solution	−20°C until use	The concentrations of GFs in the PRP and AS were not statistically different. PRP could be an effective, novel treatment option for chronic ocular surface disease.	[70]

20% AS versus conventional artificial tears treatment	Double masked randomized crossover clinical trial	2012	12	Severe DES	2 h at RT	3500 rpm/5 min at 4°C	Sterile saline solution	−20°C	AS achieves better symptom improvement compared to artificial tears in a short-term treatment.	[79]

AS: autologous serum; RT: room temperature; CBS: cord blood serum; AMT: amniotic membrane transplantation; PED: persistent epithelial defects; PK: penetrating keratoplasty; DES: dry eye syndrome, SS: Sjogren's syndrome; PRP: platelet rich plasma; GFs: growth factors.

**Table 3:** Ongoing controlled clinical studies on serum eye drops from ClinicalTrials.gov.

Trial registration number	Study type	Patient number	Blood derivatives	Conditions	End-point	Phase
NCT01089985	Interventional	10	AS	Xerophthalmia	Efficacy and safety of AS.	Phase 1

NCT01972438	Randomized Intervention Double-blind	44	Autologous serum versus saline solution	HSCT patients with severe ocular GVHD	Treatment of severe chronic ocular GVHD in HSCT patients unresponsive to standard medical treatment.	Phase 1 Phase 2

NCT01075347	Randomized Intervention Assignment Single-blind	165	Human autoserum versus traditional medications (0.1% betamethasone, 0.3% gentamicin, and 0.4% tropicamide eye drops)	Corneal epithelial defect, diabetic retinopathy, penetrating keratoplasty	Corneal epithelial healing time by slit-lamp examination with fluorescein staining.	Phase 1

NCT00681642	Observational	7	Human autoserum versus cord blood serum	Corneal epithelial defect, dry eye syndrome	Wound healing, cell proliferation and migration by means of wound healing assay evaluation, MTS assay, and Boyden chamber migration assay.	Completed

NCT00442273	Interventional Nonrandomized Double-blind	48	20% autologous serum and umbilical cord serum eye drops	Severe dry eye syndrome	Therapeutic effect between autologous serum and umbilical cord serum eye drops in the treatment of severe dry eye syndrome.	Not provided

NCT00598299	Observational Prospective	100	Human autoserum versus cord blood serum	Corneal epithelial defect, dry eye syndrome	Corneal wound healing assay evaluation, MTS assay, and Boyden chamber migration assay	Completed

NCT01168375	Intervention	80	Umbilical cord serum	Corneal epithelial defect following diabetic vitrectomy	Measurement of corneal epithelial defect in days 3, 5, 7, and 12 by slit lamp.	Phase 1

NCT01016158	Interventional Randomized Single-blind	NP	20% umbilical cord serum eye drops	Recurrent corneal erosion	Efficacy 20% umbilical cord serum eye drops.	Completed

NCT01234623	Randomized	30	Cord blood serum	GVHD, Sjogren's Disease	Healing of corneal epithelial defects, ameliorating the painful subjective symptoms.	Phase 1

NCT02291731	Interventional Single-blind	Not provided	20% autologous serum eye drops and silicone-hydrogel contact lens (CLs)	Corneal diseases	Clinical effect of combination of topical 20% autologous serum eye drops and CLs in the treatment of recalcitrant PEDs and the recurrence rate of epithelial breakdown with or without continued use of autologous serum eye drops for 2 weeks after total reepithelialization.	Currently recruiting

NCT01122303	Observational	20	20% autologous serum eye drops	SJS, nonautoimmune dry eye	Comparisons of the concentrations of EGF, TGF-*β*1, TGF-*β*2, and fibronectin in 20% AS between SJS patients with dry eye and nonautoimmune dry eye patients.	Unknown

NCT00238862	Randomized single group Open label	180	Autologous serum 20% versus amniotic membrane transplantation	PED	Corneal reepithelialization, persistent corneal reepithelialization.	Completed

NCT02153515	Single group Open label	60	Autologous serum finger prick of blood	Dry eyes, PED, ulcers	Ulcers time healing (within 4 weeks) Improving of corneal and conjunctival staining, Schirmer's test, tear break-up time, or symptoms ocular comfort index questionnaire (dry eyes).	Phase 3

NCT00779987	Interventional study Double-blind	12	20% autologous serum solution	Dry eye	Score reduction in the OSDI between patients treated with autologous serum and conventional artificial tears.	Phase 2

HCST: hematopoietic stem cell transplant; GVHD: chronic graft versus host disease; CLs: silicone-hydrogel contact lens; CLs: silicone-hydrogel contact lens; PED: persistent epithelial defects; SJS: Steven Johnson Syndrome; OSDI: Ocular Surface Disease Index; EGF: epidermal growth factor; TGF-*β*1: transforming growth factor *β*1; TGF-*β*2: transforming growth factor *β*2; MTS: 3-(4,5-dimethylthiazol-2-yl)-5-(3-carboxymethoxyphenyl)-2-(4-sulfophenyl)-2Htetrazolium.
